# Targeting PI3K/Akt/mTOR in AML: Rationale and Clinical Evidence

**DOI:** 10.3390/jcm9092934

**Published:** 2020-09-11

**Authors:** Salihanur Darici, Hazem Alkhaldi, Gillian Horne, Heather G. Jørgensen, Sandra Marmiroli, Xu Huang

**Affiliations:** 1 Haemato-Oncology/Systems Medicine Group, Paul O’Gorman Leukaemia Research Centre, University of Glasgow, Glasgow G12 0ZD, UK; Hazemalkhaldy@gmail.com (H.A.); Gillian.Horne@glasgow.ac.uk (G.H.); Heather.Jorgensen@glasgow.ac.uk (H.G.J.); 2 Department of Biomedical, Metabolic and Neural Sciences, University of Modena and Reggio Emilia, 41124 Modena, Italy; Sandra.Marmiroli@unimore.it

**Keywords:** AML, LSC, PI3K/Akt, mTOR, targeted therapy, drug resistance, combination treatment strategy

## Abstract

Acute myeloid leukemia (AML) is a highly heterogeneous hematopoietic malignancy characterized by excessive proliferation and accumulation of immature myeloid blasts in the bone marrow. AML has a very poor 5-year survival rate of just 16% in the UK; hence, more efficacious, tolerable, and targeted therapy is required. Persistent leukemia stem cell (LSC) populations underlie patient relapse and development of resistance to therapy. Identification of critical oncogenic signaling pathways in AML LSC may provide new avenues for novel therapeutic strategies. The phosphatidylinositol-3-kinase (PI3K)/Akt and the mammalian target of rapamycin (mTOR) signaling pathway, is often hyperactivated in AML, required to sustain the oncogenic potential of LSCs. Growing evidence suggests that targeting key components of this pathway may represent an effective treatment to kill AML LSCs. Despite this, accruing significant body of scientific knowledge, PI3K/Akt/mTOR inhibitors have not translated into clinical practice. In this article, we review the laboratory-based evidence of the critical role of PI3K/Akt/mTOR pathway in AML, and outcomes from current clinical studies using PI3K/Akt/mTOR inhibitors. Based on these results, we discuss the putative mechanisms of resistance to PI3K/Akt/mTOR inhibition, offering rationale for potential candidate combination therapies incorporating PI3K/Akt/mTOR inhibitors for precision medicine in AML.

## 1. Introduction

Acute myeloid leukemia (AML) is a highly heterogeneous hematopoietic malignancy, characterized by excessive proliferation and accumulation of immature myeloid blasts in the bone marrow [1]. While reductions of bulk malignant cells can be achieved in the majority of patients by standard chemotherapy consisting of cell cycle active drugs, such as cytarabine and anthracyclines, approximately two-thirds of patients relapse after the induction therapy, highlighting an unmet need for a more targeted therapeutic approach [2]. A rare population of therapy-resistant cells are believed to be the origin of relapse, termed leukemia stem cells (LSCs), also referred to as leukemia-initiating cells (LICs) [3,4,5]. These cells acquire enhanced self-renewal capacity and exhibit a block in differentiation. Thus, eliminating LSCs may prevent relapse, ultimately improving the currently poor outcome of AML. Designing LSC-targeted therapies is, however, challenging as LSCs share similar characteristics with normal hematopoietic stem cells (HSCs), making it difficult to kill LSCs whilst sparing HSCs [6,7]. In AML, dysregulated pathways affect the cellular functions of LSCs, which may represent putative targets to overcome resistance to therapy in AML [8,9].

The phosphatidylinositol-3-kinase (PI3K)/Akt and the mammalian target of rapamycin (mTOR) signaling pathway emerges as a promising therapeutic candidate to sensitize LSCs to chemotherapy. It plays an important role in both normal and malignant hematopoiesis; components of this pathway govern the expression of genes and proteins essential for cell proliferation, differentiation, and survival. Constitutive activation of PI3K/Akt/mTOR pathway is detected in 50–80% of AML patients, associated with decreased overall survival (OS) [10,11,12]. Mutations in receptor tyrosine kinases (RTKs) or GTPases are the major causes leading to upregulation of the PI3K/Akt/mTOR pathway in AML [13]. One important mechanism leading to deregulation of PI3K/Akt/mTOR signaling is mutation of fms-like tyrosine kinase 3 (FLT3). Among them, internal tandem duplication (ITD) of *FLT3* gene (FLT3-ITD) is one of the most frequent mutations in normal karyotype AML (approximately 25%). In recent clinical studies, few patients display prolonged remissions with RTK inhibitors, such as FLT3 inhibitors, highlighting the need for novel and/or partner targeted therapies [14,15]. Targeting the PI3K/Akt/mTOR pathway may be an option for FLT3-ITD AML patients.

Hyperactivation of PI3K/Akt/mTOR has also been associated with attenuated sensitivity to chemotherapy. Several studies have demonstrated that PI3K/Akt/mTOR inhibition may preferentially target LSCs. For example, the PI3K/Akt/mTOR pathway may regulate LSC survival through nuclear factor kappa-light-chain-enhancer of activated B cells (NF-κB). The pro-inflammatory transcription factor NF-κB, has been found to be aberrantly activated in LSCs but is not expressed in normal human CD34+ progenitor cells [16,17,18]. NF-κB is known to mediate chemoresistance by upregulation of anti-apoptotic genes, which enable cells to increase proliferation and evade apoptosis [19,20,21]. Targeting NF-κB may be selective for LSCs and/or sensitize LSCs to chemotherapy. Notably, NF-κB is a downstream target of PI3K/Akt/mTOR, and this signaling cascade can trigger NF-κB activation, which suggests a common survival pathway for LSCs. Treatment of AML patient samples with PI3K inhibitor LY294002 displayed inhibited Akt phosphorylation and NF-κB DNA-binding activity [22]. Furthermore, PI3K/Akt/mTOR inhibition induced apoptosis in primary AML cells and potentiated response to cytotoxic chemotherapy, while sparing normal HSC function, [23,24,25,26]. The LSC population was targeted by PI3K-directed therapies demonstrated by reduced engraftment ability of these cells in nonobese diabetic/severe combined immunodeficiency (NOD/SCID) mice [23,24]. PI3K/Akt/mTOR inhibitors may additionally potentiate LSC kill by synergizing with LSC-directed therapies. An essential feature of quiescent AML-LSCs is that they have relative lower production of reactive oxygen species (ROS) compared with bulk cells [27]. These ROS-low LSCs were shown to aberrantly overexpress Bcl-2, making them more susceptible to eradication by small-molecule Bcl-2 inhibitors like venetoclax. The therapeutic potential of venetoclax could be enhanced by PI3K/Akt/mTOR inhibition through Mcl-1-dependent mechanisms, which is a well-known determinant of resistance to venetoclax [28].

Growing evidence signposts PI3K as a druggable target for AML; indeed, there has been very productive development of small-molecule inhibitors targeting the PI3K/Akt/mTOR pathway. While PI3K/Akt/mTOR inhibitors have been effective treating other hematological malignancies, such as chronic lymphoblastic leukemia (CLL) and follicular lymphoma (FL), in AML, the clinical potential of PI3K/Akt/mTOR inhibitors has not yet been fully elucidated [29,30,31]. Clinical studies using PI3K/Akt/mTOR inhibitors as monotherapy have shown limited therapeutic efficacy, likely due to compensatory activation of other survival pathways [32]. Therefore, dissecting these relevant clinical findings and understanding how other signaling pathways impinge on PI3K/Akt/mTOR signaling pathway activity may provide us new clues as to how to effectively inhibit this pathway with potential candidate combination strategies to eradicate LSCs and so cure AML.

## 2. The PI3K/Akt/mTOR Signaling Pathway

### 2.1. Regulation of the PI3K/Akt/mTOR Pathway in Normal Hematopoiesis

The PI3K family consists of three distinct classes of PI3Ks (I-III), of which class I is implicated in regulation of hematopoiesis. Class I PI3K can be further divided into class IA and class IB enzymes, both of which are activated by cell surface receptors. Class IA PI3K can be activated by RTKs, G protein-coupled receptors (GPCRs), and oncoproteins such as the small G protein Ras, whereas class IB PI3K can be activated by GPCRs only [33,34]. Class IA PI3Ks form heterodimers between one of three catalytic subunits (p110α, p110β, or p110δ) and a regulatory adaptor molecule (p85α (or its splice variants p55α and p50α), p85β or p55γ) [35,36]. Each pair shares some overlap whilst maintaining distinct function. In contrast to the heterogeneity of class IA, a single class IB isoform has been described that associates catalytic subunit p110γ with regulatory adaptor molecule p101 or p84 [37,38]. While catalytic subunits p110α and p110β are consistently expressed in a broad range of tissues, p110γ and p110δ are specifically enriched within the hematopoietic system—preferentially in leukocytes [39].

**Figure 1:**
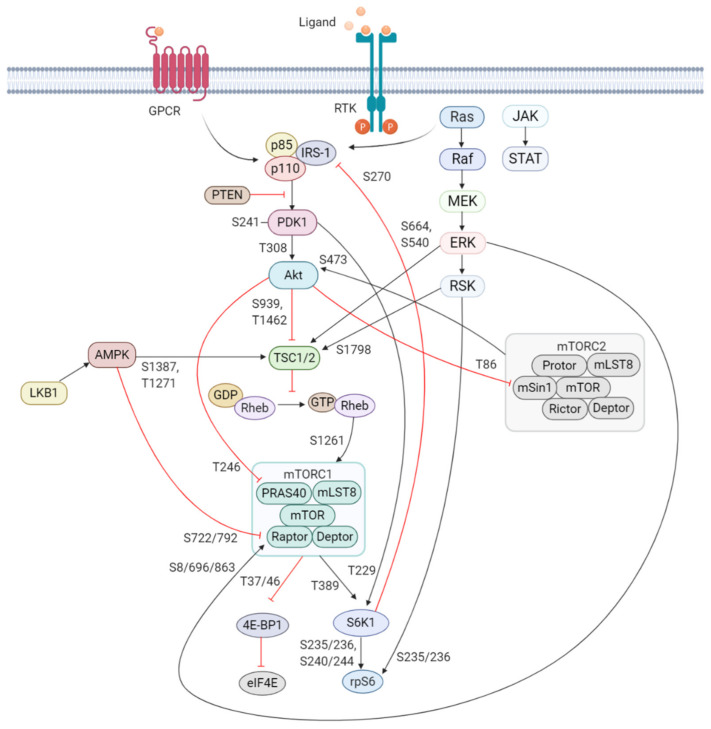
Schematic overview of the activation and regulation of the PI3K/Akt/mTOR signaling pathway. Activation of PI3K is stimulated by binding of an extracellular ligand (e.g., hormones, growth factors, and cytokines) to a cell surface receptor such as the receptor tyrosine kinase (RTK) in the plasma membrane. Activated RTK recruits adaptor proteins, which bind to the regulatory p85 subunit of PI3K and subsequently activate the catalytic subunits for full PI3K activation. PI3K is also activated by G protein-coupled receptors (GPCR) or small GTPase Ras, which bind PI3K directly. Activated PI3K catalyzes the phosphorylation of phosphatidylinositol-4,5-phosphate (PIP_2_) to generate phosphatidylinositol-3,4,5-phosphate (PIP_3_). PIP_3_ recruits phosphoinositide-dependent kinase 1 (PDK1) and Akt to the plasma membrane inducing Akt phosphorylation by PDK1 at T308. Akt activation is completed by phosphorylation at S473 by mTOR complex 2 (mTORC2). The mTOR complex includes two distinct protein complexes, mTORC1 and mTORC2. mTORC1 comprises of mTOR, proline-rich Akt substrate 40 kDa (PRAS40), regulatory-associated protein of mTOR (Raptor), mammalian lethal with Sec13 protein 8 (mLST8, also known as GβL), and DEP-domain-containing mTOR-interacting protein (Deptor) [44]. mTORC2 comprises of mTOR, mLST8, Deptor, protein observed with Rictor-1 (Protor), rapamycin-insensitive companion of mTOR (Rictor), and mammalian stress-activated protein kinase interacting protein (mSin1) [45]. Akt indirectly activates mTORC1 by phosphorylation and inhibition of tuberous sclerosis complex 2 (TSC2) at S939 and T1462, releasing the inhibitory effects of this complex on Ras-related GTPase Rheb, an activator of mTORC1. Akt also directly controls activation of mTORC1 in a TSC2-independent manner via phosphorylation of PRAS40 at T246. The extracellular signal-regulated kinase (ERK)/90 kDa ribosomal S6 kinase (RSK) and liver kinase B1/AMP-activated protein kinase (LKB1/AMPK) signaling pathways impinge on several nodes of the PI3K/Akt/mTOR pathway and can modulate mTORC1 activity. Both ERK and RSK modulate mTORC1 activity by phosphorylation of TSC2 at S664 and S540 (ERK) and S1798 (RSK). ERK1/2 can also control mTORC1 activation by phosphorylation of Raptor at S8, S696, and S863. Master metabolic regulator AMPK inhibits mTORC1 activity in two different pathways, the first by phosphorylation of TSC2 at T1271 and S1387 and the second by phosphorylation of Raptor at S722 and S792. Activated mTORC1 promotes cap-dependent mRNA translation via phosphorylation of eukaryotic translation initiation factor 4E (eIF4E)-binding protein 1 (4E-BP1) at T37 and T46, which is a priming event required for subsequent phosphorylation of several carboxy-terminal serum-sensitive sites to release 4E-BP1 from eIF4E. Ribosomal protein S6 kinase beta-1 (S6K1) is a downstream target of mTORC1, activated by phosphorylation at T389 by mTORC1 as well as T229 phosphorylation mediated by PDK1. S6K1 in turn activates ribosomal protein S6 (rpS6), which is dispensable for cell growth and protein synthesis. RSK can also directly activate rpS6 via phosphorylation at S235 and S236. The black arrows represent positive regulation (activation), whereas the red blunt-ended lines indicate negative regulation (inhibition). IRS-1 = insulin receptor substrate 1, PTEN = phosphatase and tensin homolog, GDP = guanosine diphosphate, GTP = guanosine triphosphate, JAK = Janus kinase, STAT = signal transducer and activator of transcription. Created with BioRender.com.

In response to extracellular stimuli (e.g., hormones, growth factors, and cytokines) and the subsequent activation of RTKs, class IA PI3K is recruited to the plasma membrane via interaction of p85 with adaptor proteins, such as insulin receptor substrate (IRS) 1/2 or growth factor receptor-bound protein 2-associated binding protein 2 (GAB2) that bind to the regulatory p85 subunit of PI3K [40,41]. The class IB p110γ is activated by GPCRs through direct interaction of its regulatory adaptor molecule with Gβγ subunit of trimeric G proteins [38]. The activated p110 catalytic subunit facilitates the phosphorylation of phosphatidylinositol-4,5-phosphate (PIP_2_) to generate phosphatidylinositol-3,4,5-phosphate (PIP_3_). PIP_3_ recruits phosphoinositide-dependent kinase 1 (PDK1) and Akt/protein kinase B (PKB) to the plasma membrane where PDK1 phosphorylates Akt at Threonine(T)308 residue within the activation loop of the kinase domain to initiate the activation of Akt [42,43] (Figure 1).

**Figure 2:**
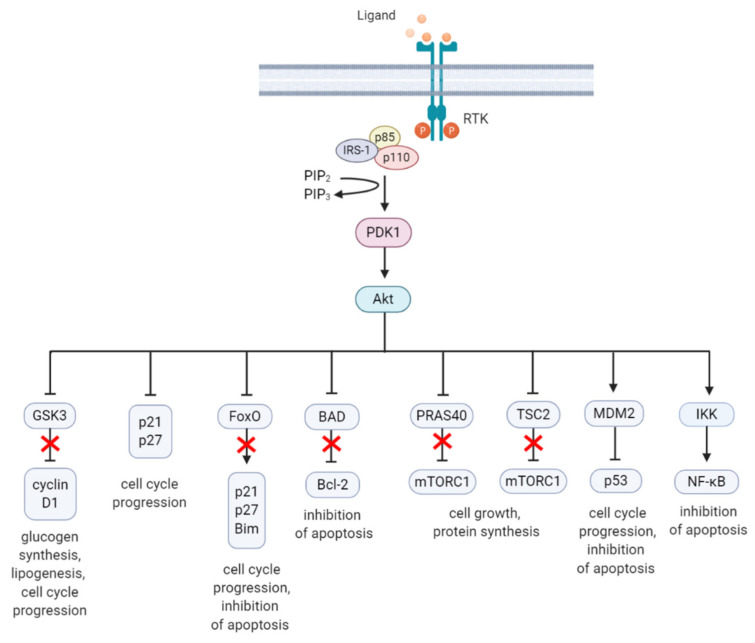
A summary diagram of Akt downstream target molecules. (From left to right) Fully activated Akt controls numerous effectors implicated in cell growth, proliferation, differentiation, metabolism, and survival, of which some are highlighted. Akt regulates G1/S cell cycle progression by phosphorylation and inactivation of GSK3/cyclin D1, p21, and p27. Akt was found to regulate cell metabolism by mediating lipogenesis and glucose uptake through phosphorylation and inhibition of GSK3, which inhibits glycogen synthesis. Akt controls apoptosis by phosphorylation and inhibition of FoxO and pro-apoptotic Bcl-2 family member BAD. Akt promotes cell growth by activation of mTORC1 though phosphorylation of PRAS40, which prevents its inhibition of mTORC1. Akt can also induce mTORC1 activation through phosphorylation and inhibition of TSC2, relieving the inhibitory effects of the TSC1/TSC2 complex on mTORC1. Akt enhances MDM2-mediated ubiquitination and proteasomal-dependent degradation of p53. Akt can inhibit apoptosis and promote cell survival by activating NF-κB. The arrows represent positive regulation (induction/activation), whereas the blunt-ended lines indicate negative regulation (inhibition/inactivation). The red cross represents inhibition caused by Akt-mediated negative regulation. RTK = receptor tyrosine kinase, IRS-1 = insulin receptor substrate 1, PIP_2_ = phosphatidylinositol-4,5-phosphate, PIP_3_ = phosphatidylinositol-3,4,5-phosphate, PDK1 = phosphoinositide-dependent kinase-1, MDM2 = mouse double minute 2 homolog, GSK3 = glycogen synthase kinase 3, TSC2 = tuberous sclerosis complex 2, mTORC1 = mTOR complex 1, FoxO = forkhead box O, Bim = Bcl-2-like protein 11, BAD = BCL-2 associated agonist of cell death, Bcl-2 = B-cell lymphoma 2, IKK = IκB kinase, NF-κB = nuclear factor kappa-light-chain-enhancer of activated B cells. Created with BioRender.com.

Akt is a highly conserved serine/threonine kinase that has multiple diverse functions. Full Akt activation, in addition to PDK1-mediated phosphorylation, requires phosphorylation at Serine(S)473 residue in the regulatory domain, by mTOR complex 2 (mTORC2), integrin-linked kinase (ILK), PDK1 or members of the PI3K-related kinase (PIKK) family, such as DNA-dependent protein kinase (DNA-PK) [46,47]. Notably, Akt can activate mTORC2 through a positive feedback loop by direct phosphorylation of mTORC2 component mammalian stress-activated protein kinase interacting protein (mSin1) at T86 [48,49]. Activated Akt can phosphorylate a wide spectrum of protein substrates, including forkhead box class O (FoxO), glycogen synthase kinase-3 (GSK3) α/β, and Bcl-2 associated agonist of cell death (BAD), maintaining cell cycling, survival, metabolism, cell growth and other essential cellular functions (Figure 2) [50].

Regulation of PI3K activity is negatively controlled by the tumor suppressor phosphatase and tensin homolog (PTEN) and Src homology domain-containing inositol phosphatase (SHIP) to maintain normal hematopoiesis. PTEN is a lipid phosphatase that hampers PI3K signaling through dephosphorylation of the lipid signaling intermediate PIP3 [51]. Loss of function of *PTEN* through mutations, genetic silencing, or epigenetic mechanism is implicated in the pathology of multiple human malignancies and can lead to aberrant PI3K/Akt/mTOR signaling [52]. SHIP is predominantly expressed in hematopoietic cells and hydrolyzes PIP_3_ to generate PI(3,4)P_2_ [53]. Mutation of the *SHIP* gene was detected in a low percentage of AML and acute lymphoblastic leukemia (ALL) patients [54].

One of the key downstream targets of the Akt is mTOR, which positively regulates cell growth and proliferation by promoting protein synthesis and inhibition of autophagy [55,56]. The identification of this serine/threonine kinase stems from the discovery of the natural product rapamycin, originally extracted from the soil bacterium *Streptomyces hygroscopicus* [57]. mTOR participates in two functionally distinct multiprotein complexes, mTORC1 and mTORC2, of which only mTORC1 is sensitive to inhibition by rapamycin [58]. Akt indirectly activates mTORC1 by phosphorylation of tuberous sclerosis complex 2 (TSC2) at S939 and T1462 [59,60]. TSC2 functions with TSC1 forming a heterodimeric complex which blocks the activation of Ras homolog enriched in brain (Rheb). Akt phosphorylation of TSC2 inhibits the GTPase-activating protein (GAP) activity of this complex and in turn permits Rheb to activate mTORC1. Akt can also activate mTORC1 by a TSC2-independent mechanism which involves phosphorylation of proline-rich Akt substrate 40kDa (PRAS40), a component of mTORC1. Phosphorylation of PRAS40 at T246 results in dissociation of PRAS40 from mTORC1 and attenuates the inhibitory effect of PRAS40 on mTORC1 activity [61]. Upon activation, mTORC1 is phosphorylated on several residues (T2446, S2448, and S2481), but no function has been ascribed to any phosphorylation site [62,63,64]. S1261 was identified as a site-specific phosphorylation site of mTORC1 that in response to insulin signals via the PI3K/TSC2/Rheb axis regulating mTORC1 function in an amino acid-dependent and rapamycin-insensitive mechanism.

The highly conserved protein kinase mTOR is a central hub of nutrient signaling and cell growth and integrates multiple intracellular signals [65]. With respect to the mTOR signaling pathway, the TSC1/TSC2 complex has emerged as a sensor and integrator of multiple signaling pathways to modulate mTORC1 activity [66] (Figure 1). One important signaling pathway that negatively regulates mTORC1 activity is the liver kinase B1 (LKB1)/ AMP-activated protein kinase (AMPK) pathway [67]. AMPK is a cellular energy sensor activated in various conditions that deplete cellular energy, such as nutrient deprivation or hypoxia [68,69]. Phosphorylation of AMPK at T172 in the activation loop is required for its kinase activity and is mediated by LKB1, the upstream serine/threonine kinase of AMPK [70]. AMPK inhibits mTORC1 in two different ways, i.e., phosphorylation of TSC2 at T1227 and S1345; and phosphorylation of Raptor at S722/792, which is a component of mTORC1 [71,72]. In addition to AMPK, the extracellular signal-regulated kinase (ERK)/ p90 ribosomal S6 kinase (RSK) pathway also modulates mTORC1 activity. ERK/RSK is one of the other main signaling networks activated in parallel with PI3K by RTKs to control survival, differentiation, proliferation, and metabolism [73]. Both ERK and RSK promote mTORC1 activity by phosphorylation of TSC2 at ERK S664 and S540, and RSK S1798 [74,75]. In addition, ERK1/2 contributes to Ras-dependent activation of mTORC1 through phosphorylation of Raptor at S8, S696, and S863 [76].

Upon activation, mTORC1 phosphorylates its main downstream targets eukaryotic initiation factor-4E (eIF4E) -binding protein 1 (4E-BP1) and rapamycin-sensitive ribosomal protein S6 kinase beta-1/p70 ribosomal S6 kinase (S6K1), involved in the translation of mRNAs. 4E-BP1 inhibits the initiation of cap-dependent translation by binding and inactivating eIF4E. This binding is reversible, and mTORC1 phosphorylation of 4E-BP1 at T37/46 relieves 4E-BP1 from eIF4E [77]. Released eIF4E assembles at the 5′ end of mRNA, which facilitates the recruitment of the ribosome and subsequent initiation of translation [78].

S6K1 is the other main target of mTORC1 implicated in the regulation of cell growth. Activation of S6K1 requires phosphorylation at T229 and T389, of which T229 is phosphorylated by PDK1 and T389 by mTORC1 [79,80,81]. Activated S6K1 activates 40S ribosomal protein (rp) S6 that represents the most extensively studied substrate of S6K1. rpS6 becomes phosphorylated on several serine residues [82]. While S6K1 phosphorylates rpS6 on all phosphorylation sites (S235/236 and S240/244), RSK phosphorylates rpS6 exclusively on S235/236 in an mTOR-independent mechanism, suggesting that ERK/RSK pathway contributes to rpS6 phosphorylation upon mitogen stimulation [83]. mTORC1-stimulated S6K1 mediates an important negative feedback regulation of PI3K through phosphorylation of IRS-1. As such, S6K1 phosphorylates IRS-1 proteins at several serine residues (S270, S307, S636, and S1101) of which S270 was found to be required for S6K1/IRS-1 interaction and subsequent phosphorylation of the other S6K1-specific residues [84]. Phosphorylation of IRS-1 induces its protein degradation and insulin resistance, thereby inhibiting the insulin-like growth factor 1 (IGF-1) -mediated PI3K activation.

### 2.2. Constitutive PI3K/Akt/mTOR Activation in AML

Regulated PI3K/Akt/mTOR signaling is critical for normal hematopoiesis, with deregulation of PI3K/Akt/mTOR activity linked to depletion of HSC pool [85]. For example, *Pten* deletion in adult mice HSCs activated the PI3K/Akt/mTOR pathway and promoted HSC proliferation and depletion through induced expression of p16^Ink4a^ and p53, and leukemogenesis [86,87,88]. These effects were mostly mediated by mTOR as rapamycin was able to suppress leukemogenesis and restore normal HSC function [88]. Myristoylated Akt1 (myr-Akt) was introduced into HSCs via retroviral transduction of bone marrow cells and subsequent transplantation, to mimic constitutively active Akt, which is frequently observed in AML [85]. Results revealed that myr-Akt contributes to myeloproliferative disorders (MPD), and T-cell lymphoma with high frequency, and AML with a lower penetrance. HSCs in the myr-Akt1 mice displayed transient expansion of immature myeloid cells in the bone marrow and spleen, and increased cycling, associated with impaired engraftment. The importance of mTOR signaling as a mediator of Akt was demonstrated with rapamycin. Rapamycin rescued cobblestone formation in myr-Akt–transduced bone marrow cells in vitro and increased survival of myr-Akt mice. TSC1 is also potentially involved in leukemogenesis. Deletion of *TSC1* in HSCs leads to activation of mTOR signaling, causes rapid HSC cycling and elevated levels of ROS, and impaired HSC self-renewal [89]. Importantly, treatment with a ROS antagonist in vivo demonstrated that the TSC1/mTOR axis is important to maintain HSC quiescence and function by suppressing ROS. These findings indicate that mTOR is an important mediator of PI3K regulation in HSCs. Furthermore, FoxO transcription factors are functionally redundant in HSC homeostasis through regulation of HSC response to physiologic oxidative stress, quiescence, and survival [90,91,92]. Mice engineered with conditional knockout alleles of *Foxo1*, *Foxo3*, and/or *Foxo4* displayed increased cell cycling and apoptosis of HSC, and a marked increase in ROS levels.

The PI3K/Akt/mTOR signaling pathway is frequently hyperactivated in AML cells and potentially contributes to uncontrolled growth, proliferation, differentiation, metabolism, and survival [10,93]. The PI3K/Akt/mTOR pathway is also important for the regulation of the AML-LSC population, demonstrated in mouse models with genetic alterations of key PI3K/Akt/mTOR signaling genes. Rheb1 is overexpressed in AML patients, which was associated with reduced survival in comparison to patients with lower Rheb1 expression [94,95,96]. Deletion of *Rheb1* induced apoptosis and enhanced cell cycle arrest in LSCs, and prolonged survival of MLL-AF9-induced leukemic mice, suggesting that the mTORC1 pathway may be required for LSC maintenance [94]. PDK1 is overexpressed in over 40% of AML patients and is required for Akt activation [97]. Deletion of *Pdk1* in MLL-AF9-induced mice resulted in a reduction of LSCs and upregulated the expression of apoptosis inducers, such as *BAX* and *Tp53* [98,99]. Mice transplanted with MLL-AF9-positive LSCs were also shown dependent on S6K1 for LSC maintenance [95]. Loss of S6K1 improved survival of MLL-AF9-induced leukemic mice, which was associated with reduced Akt and 4E-BP1 phosphorylation. Furthermore, the PI3K/Akt/mTOR pathway is also implemented in the crosstalk between LSCs and the stromal cells associated with its niche that promotes the drug-resistant phenotype of LSCs. Several reports have demonstrated that pharmacological inhibition of PI3K/Akt/mTOR signaling may effectively target leukemic cells within the bone marrow niche [8,100,101].

**Figure 3:**
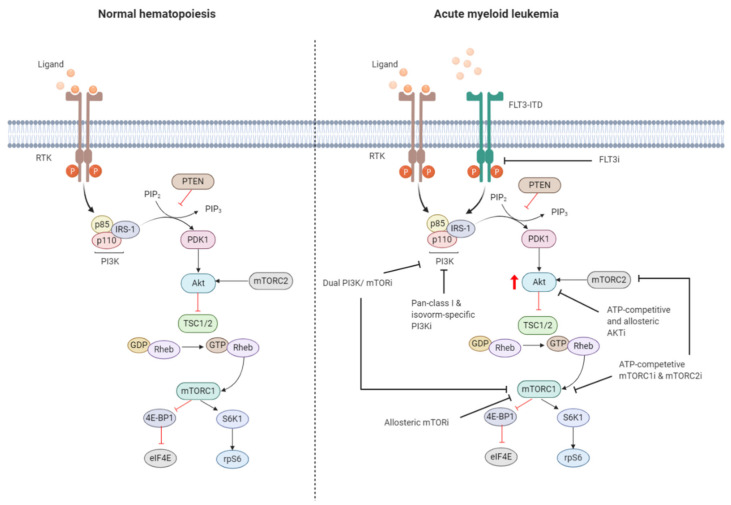
Targeting the PI3K/Akt/mTOR pathway in AML. The PI3K/Akt/mTOR pathway is commonly dysregulated in AML caused by mutations in membrane-bound proteins such as receptor tyrosine kinases (RTKs) and small GTPase Ras. Activating mutations in fms-like tyrosine kinase 3 (FLT3), such as the FLT3-internal tandem duplication (FLT3-ITD), are an important mechanism leading to dysregulation of PI3K/Akt/mTOR signaling. The ITD mutation causes ligand-independent activation of the FLT3 receptor, leading to constitutive activation of the PI3K/Akt/mTOR pathway. Numerous small-molecule inhibitors of this pathway include FLT3 inhibitors (FLT3i), dual PI3K/mTORi, allosteric mTORi, pan-class I and isoform-specific PI3Ki, ATP-competitive and allosteric Akti, and ATP-competitive mTOR complex 1 (mTORC1) and mTOR complex 2 (mTORC2) inhibitors (mTORC1i and mTORC2i). The red arrow indicates elevated Akt phosphorylation, whereas the red blunt-ended lines represent negative regulation (inhibition). IRS-1 = insulin receptor substrate 1, PIP_2_ = phosphatidylinositol-4,5-phosphate, PIP_3_ = phosphatidylinositol-3,4,5-phosphate, PTEN = phosphatase and tensin homolog, PDK1 = phosphoinositide-dependent kinase-1, TSC 1/2 = tuberous sclerosis complex 1/2, ATP = adenosine triphosphate, GDP = guanosine diphosphate, GTP = guanosine triphosphate, Rheb = Ras homolog enriched in brain, 4E-BP1 = eukaryotic translation initiation factor 4E (eIF4E)-binding protein 1, S6K1 = ribosomal protein S6 kinase beta-1, rpS6 = ribosomal protein S6. The black blunt-ended lines indicate the main targets for therapeutic intervention. Created with BioRender.com.

Dysregulation of the PI3K/Akt/mTOR pathway is often the result of loss or inactivation of tumor suppressors, mutation or amplification of PI3K, as well as activation of RTKs or oncoproteins upstream of PI3K (Figure 3) [102,103]. About 50–80% of patients with AML display constitutive PI3K/Akt/mTOR activation, and this was associated with significant poorer OS [104]. Poor prognosis in AML patients with constitutive PI3K/Akt/mTOR signaling could be related to the fact that this pathway is associated with chemoresistance, which contributes to the short-term survival in AML [25,105,106]. However, no correlation was shown to exist between PI3K/Akt/mTOR activity and a particular AML subtype, cytogenetic abnormality, or etiology of the disease [107,108].

Exploring mechanisms of constitutive PI3K/Akt/mTOR activation in AML identified mutations of RTKs (e.g., FLT3-ITD, c-KIT) or GTPases (KRAS, NRAS) as well as autocrine IGF-1/IGF-1R signaling responsible for dysregulation [109,110]. Aberrant PI3K/Akt/mTOR signaling activation is often associated with enhanced Akt phosphorylation, mediated by phosphorylation at S473 by PDK1 and T308 by mTORC2. The OS of AML patients presenting with Akt phosphorylation at these sites was found to be significantly shorter in several studies [104,106]. Furthermore, mTORC1 is activated in most AML patients, indicated by enhanced phosphorylation of its main downstream substrates 4E-BP1, S6K1, and rpS6 [24,111]. However, activation of mTORC1 and its downstream target may also occur independently of PI3K/Akt though parallel signaling pathways [111,112,113]. It is therefore important to dissect how PI3K/Akt/mTOR signaling converges with other signaling pathways, which may have clinical implications for selection of drugs targeting different signaling molecules.

About 30% of AML patients with normal karyotype present with an activating FLT3 receptor mutation, most often as FLT3-ITD, and is the major intercessory of PI3K/Akt/mTOR pathway dysregulation. FLT3 is a member of the class III RTK family and is important for the maintenance of hematopoietic homeostasis [114,115,116]. FLT3-ITD exhibits ligand-independent constitutive tyrosine kinase activity and activates signaling pathways including PI3K/Akt/mTOR [117]. However, regulatory p85 subunit of PI3K does not bind to the FLT3 receptor, nor is it tyrosine phosphorylated after FLT3 ligand stimulation. Instead, p85 associated with SH2 domain-containing protein tyrosine phosphatase-2 (SHP-2) and SHIP, in murine Ba/F3 cells stably transfected with human FLT3-ITD [118]. FLT3-ITD expression in Ba/F3 cells was associated with constitutive activation of Akt and concomitant phosphorylation of FoxO3a [110,119]. FoxO3a has an important role in apoptosis and cell cycle regulation [120]. FLT3-ITD was shown to constitutively activate Akt, and concomitantly phosphorylate FoxO3a, suppressing the expression of FoxO3a target genes encoding for p27 and pro-apoptotic Bcl-2 family member, Bim [110,121]. FLT3-ITD negatively regulates FoxO3a, thereby suppressing FoxO3a-mediated apoptosis and bypassing the G1 cell cycle blockade.

### 2.3. Targeting the PI3K/Akt/mTOR Signaling Pathway in AML

Preclinical evidence underlines the significant role of PI3K/Akt/mTOR signaling in leukemia initiation and maintenance. There is considerable interest targeting PI3K/Akt/mTOR signaling for AML treatment, which has resulted into the rapid development of small molecule compounds that target either a single or multiple kinase (Figure 3). PI3K-Targeting molecules can be divided into isoform-specific PI3K inhibitors and ATP-competitive pan-PI3K inhibitors. The PI3K p110δ catalytic subunit is consistently expressed at a high level in AML blasts, making it an attractive therapeutic target for AML [122]. Idelalisib (also referred to as CAL-101), for example, is a p110δ inhibitor that is currently under Phase 3 clinical investigation for the treatment of B-cell malignancies [123]. Treatment of AML cells with idelalisib inhibited ribosomal RNA (rRNA) synthesis and cell proliferation by suppressing Akt phosphorylation with a greater effect observed in cells expressing higher levels of p110δ [124]. Pan-PI3K inhibitors target all isoforms of PI3K and may exert broader anti-leukemic effects but at the expense of higher toxicity. mTOR inhibitors include both ATP-pocket and allosteric mTOR binding drugs, e.g., rapalogs, such as everolimus and temsirolimus. Both drugs derive from the natural macrolide rapamycin and act by associating with immunophillin FK506 binding protein 12 (FKBP12), which in turn binds and inhibits mTORC1, although after lengthy exposure they inhibit also mTORC2 [125]. mTOR inhibitors represent the first class of PI3K/Akt/mTOR-directed therapies and yielded promising anti-proliferative effects without inhibition of normal CD34+ cells in preclinical settings [23]. The anti-leukemic effects were associated with reduced phosphorylation of S6K1 and 4E-BP1 and could be enhanced in combination with conventional cytotoxic drugs [24]. An important drawback of inhibiting mTORC1 is the increased phosphorylation of Akt. Dual PI3K and mTOR inhibitors block both the upstream and downstream targets of Akt, thereby circumventing the increased PI3K and Akt signaling subsequent to mTORC1 inhibition [126]. Dual PI3K/mTOR inhibitor dactolisib efficiently blocked PI3K and mTORC1 signaling and mTORC2 activity [127]. Furthermore, dactolisib inhibited protein translation in AML cells, reducing cell growth and inducing apoptosis without affecting survival of normal CD34+ cells. A small number of Akt inhibitors have been developed, but they are rarely evaluated in preclinical or clinical settings as the development of Akt inhibitors has long been hampered by high structural similarity of the Akt catalytic domain to that of other kinases of the AGC kinase family (named after the representative protein kinase A, G, and C families) [93,128].

## 3. Crosstalk of the PI3K/Akt/mTOR Signaling Pathway with Other Signaling Pathways in AML

We have demonstrated the complexity of the PI3K/Akt/mTOR signaling pathway resulting from several feedback loops and crosstalk with other signaling pathways that are able to modulate PI3K/Akt/mTOR signaling. In this section we will particularly focus on FLT3-ITD AML which is the major driver of dysregulation of PI3K/Akt/mTOR signaling. FLT3-ITD has been shown to induce, in addition to PI3K/Akt/mTOR, other downstream effectors, including MEK/ERK/RSK1 and Janus kinase (JAK)/signal transducer and activator of transcription (STAT) pathways, FoxO3a phosphorylation, and ROS production [129,130,131]. These effectors interact with the PI3K/Akt/mTOR pathway, elucidation of which may reveal possible mechanisms of resistance to PI3K-directed therapies. Recent studies have stressed that FLT3-ITD induces genomic instability through ROS production, which potentially contributes to chemoresistance leading to disease relapse [132,133]. The PI3K/Akt/mTOR signaling pathway is linked to both ROS production and the DNA damage response (DDR) pathway, strengthening the premise of further exploiting this pathway as a potential therapeutic target [46,134,135].

The Ras/Raf/MEK/ERK and PI3K/Akt/mTOR signaling pathways strongly regulate each other and share overlapping downstream effectors to regulate multiple cellular functions. Several mechanisms of integration of these two pathways have been identified, including negative feedback loops, cross-inhibition, cross-activation, and pathway convergence, which have been described in the previous section [73]. Briefly, the Ras/ERK pathway cross-activates PI3K/Akt/mTOR signaling by regulating PI3K, TSC2, and mTORC1. Ras-GTP can directly bind and allosterically activate PI3K [136]. Activated Ras/ERK pathway can positively regulate mTORC1 activity indirectly through phosphorylation of TSC2 or directly through PI3K-independent phosphorylation of proline-directed residues within Raptor [74,75,76]. Furthermore, Ras/ERK and PI3K/Akt/mTOR pathways co-regulate downstream effectors, including FoxO and c-Myc transcription factors, BAD, and GSK3 [137,138,139,140,141].

The JAK/STAT pathway is a core oncogenic signaling pathway activated by a plethora of cytokines and hormones and has a critical role in hematopoietic malignancies [142]. Deregulation of the JAK/STAT pathway, in particular STAT5, is frequently reported in AML and is indispensable for leukemia cell maintenance and survival [143,144]. Persistent STAT5 activity is driven by oncogenic FLT3-ITD independent of JAKs and is associated with resistance to PI3K/Akt/mTOR inhibitors [145,146,147]. This resistance mechanism involved the increased expression of PIM kinases. PIM kinases are oncogenic serine/threonine kinases overexpressed in several malignancies that promote cellular functions, including cell cycle progression, proliferation, survival, and drug resistance [148,149]. PIM-1 is transcriptionally upregulated by constitutively active FLT3 and contributes to FLT3-ITD-mediated survival [150]. Furthermore, PIM-1 phosphorylates and stabilizes FLT3, promoting FLT3 signaling in a positive feedback loop [151]. It has been reported that PIM kinases are expressed downstream of STAT5 activation in FLT3-ITD AML and are implicated in the regulation of mTORC1 to promote cap-dependent translation in parallel with the PI3K/Akt pathway [152,153]. PIM and Akt share common substrates of the apoptosis machinery and cellular metabolism which may account for PIM kinases’ ability to act as a resistance mechanism [154]. For instance, it has been reported that PIM-1 elevates RTKs upon inhibition of Akt, leading to drug resistance [155,156]. Upregulation of PIM-1 mediated by FLT3-ITD enhanced survival through protection of the mTOR/4E-BP1/myeloid cell leukemia 1 (Mcl-1) pathway, which was abrogated by inhibition of the STAT5/PIM kinase axis [157,158,159,160,161].

Both PI3K/Akt and STAT5 signaling converge to control ROS production, which is induced by FLT3-ITD. ROS are a heterogeneous group of molecules and radicals primarily produced in the electron transport chain (ETC) of the mitochondria, in particular from Complex I and III [162,163]. ROS have a critical role in the regulation of normal hematopoiesis and is commonly elevated in a wide range of human cancers, including AML [164,165]. Activating mutations of FLT3 has been shown to increase ROS production, causing DNA damage and affecting the DDR. The DNA damage is repaired by error-prone alternative non-homologous end-joining (alt-NHEJ) pathway, which promotes genomic instability and leukemogenesis [130]. Elevated ROS in FLT3-ITD AML is attributable to constitutive activation of nicotinamide adenine dinucleotide phosphate oxidases (NOX). [166,167,168]. Another proposed mechanism of FLT3-ITD-driven ROS production involves interaction of STAT5 with RAC1, a small GTPase protein, which is an essential component of NADPH oxidase [130,169]. Interestingly, ROS can increase phosphorylation of STAT5 via JAK, which acts as a feed-forward loop [169]. It was demonstrated that inhibition of FLT3-ITD not only decreased tyrosine phosphorylation of STAT5 but also reduced RAC1 activity and its binding to NOX [170].

Aberrant PI3K/Akt/mTOR signaling drives ROS production through different molecular mechanisms, including the activation of NOXs [171,172,173]. Wortmannin (inhibitor for PI3Ks and PI3K-related enzymes) treatment or *Akt1* knockout impaired translocation of NOX subunits and reduced production of ROS [173,174]. The PI3K/Akt/mTOR pathway is also involved in the regulation of the gene expression of endogenous antioxidant synthesis. ROS production is tightly regulated by the redox-sensitive signaling system Kelch-like ECH-associated protein 1 (Keap1)/nuclear factor erythroid 2-related factor 2 (Nrf2)/antioxidant response elements (ARE). Under physiological conditions, Nrf2 is sequestered in the cytosol via its association with Keap1. When ROS levels increase, the Nrf2 dissociates from Keap1 and enters the nucleus where it forms a heterodimer with a small musculoaponeurotic fibrosarcoma (MAF) protein to activate ARE-dependent gene expression of antioxidative proteins [175,176]. The PI3K/Akt/mTOR signaling pathway contributes to Nrf2-mediated regulation of antioxidative proteins as the nuclear translocation of Nrf2 requires PI3K activation [177]. PI3K inhibition using wortmannin and LY294002 was shown to inhibit Nrf2 activity [178]. As FLT3-ITD induces phosphorylation of FoxO3a (which requires the presence of Akt phosphorylation sites), it seems apparent that FLT3-ITD promotes ROS production at least in part through dysregulation of the PI3K/Akt/mTOR pathway [167,172]. Paradoxically, excessive ROS production can promote activation of PI3K/Akt/mTOR signaling through oxidative inhibition of PTEN, causing an increase in cellular PIP_3_ levels and activation of Akt [171,179,180].

## 4. Clinical Implications of PI3K/Akt/mTOR Inhibitors in AML

There is considerable interest in small-molecule drugs targeting the PI3K/Akt/mTOR pathway, of which some have been approved by the United States Food and Drug Administration (FDA) for several human cancers (Table 1). However, despite extensive pre-clinical and clinical research of these drugs either as a monotherapy or in conjunction with conventional cytotoxic chemotherapeutics, they have not yet been successfully translated into clinical practice for the treatment of AML. Here, we review PI3K/Akt/mTOR inhibitors subjected to clinical evaluation for AML therapy, summarized in Table 1 and Table 2. The response criteria for AML are summarized in Table 3.

### 4.1. Buparlisib

Buparlisib (BKM120; NVP-BKM120) is a highly selective pan-PI3K inhibitor that has shown promising activity against solid tumors as well as lymphoid malignancies. Buparlisib binds to all class IA p110 catalytic subunits and inhibits PI3K in an ATP-competitive manner [185,186]. Several reports have demonstrated that buparlisib inhibits cell proliferation, diminishes metabolic activity, and exerts cytotoxic effects on solid tumors and hematological malignancies, including AML, by selective inhibition of Akt activity [187,188,189,190]. The mechanism by which buparlisib impaired viability was associated with the induction of p21-mediated G2/M cell cycle arrest and reduced expression of NF-κB anti-apoptotic proteins [191]. In AML, buparlisib showed in vivo effectiveness in, and prolonged survival of, xenotransplant mouse models [190]. In addition, it has been demonstrated that combination with other small-molecule drugs, such as the glycolitic modulator dichloroacetate (DCA), proteasome inhibitor bortezomib, and c-Myc inhibitor 10058-F4, profoundly enhanced the cytotoxic effect of buparlisib in AML cell lines and/or primary samples [190,191].

Buparlisib was clinically evaluated by Ragon et al. in an open label, non-randomized Phase 1 dose escalation study in patients with relapsed/refractory AML (*n* = 12), ALL (*n* = 1) or mixed phenotype acute leukemia (MPAL) (*n* = 1) [192]. In the AML population, cytogenetic analysis detected diploid karyotype (*n* = 4), inversion 3 or 3q26 (*n* = 3), and other cytogenic alterations (*n* = 5). Molecular analysis revealed isocitrate dehydrogenase (IDH) and RAS mutation (*n* = 2), FLT3 D835 mutation (*n* = 1), NRAS mutation (*n* = 1), KRAS and NRAS mutation (*n* = 1), tumor protein 53 (TP53) mutation (*n* = 1), and nucleophosmin 1 (NPM1), NRAS, IDH1, DNA methyltransferase 3A (DNMT3A) mutation (*n* = 1). Three patients experienced clinically significant confusion, supported by earlier studies reporting that buparlisib crosses the blood-brain barrier and can cause neurotoxicity. Protein profiling by Western blot revealed that buparlisib was able to block PI3K/Akt/mTOR signaling, observed by decreased p-S6K1 and p-FoxO3a, and reverse phase protein array (RPPA) analysis showed significant downregulation of PRAS40. However, direct inhibition of PI3K led to Akt induction, pointing towards a compensatory feedback mechanism. These findings confirm that buparlisib can effectively inhibit and target the downstream PI3K/Akt/mTOR pathway, but such biological suppression did not translate into an improved clinical response in this patient population. Overall, only one patient (with inversion 3) had stable disease receiving maximum tolerated dose of BKM120 for a period of 82 days. Notably, three patients (21.4%) with 3q26 abnormalities had the longest OS. As patients carrying 3q26 gene rearrangements present up-regulation of PI3K/Akt/mTOR signaling and here displayed significantly improved OS, patients with 3q26 aberrations may be more sensitive to pan PI3K inhibitors.

### 4.2. Gedatolisib

Gedatolisib (PF-05212384; PKI-587) is a highly potent, selective and ATP-competitive dual inhibitor of PI3Kα, PIK3γ and mTOR for clinical development [193]. In vivo, gedatolisib has demonstrated antitumor activity in solid tumor xenograft models and has been clinically evaluated in patients with advanced cancers [194,195,196,197,198]. Gedatolisib was able to inhibit cell viability in solid tumor models and effectively targeted and inhibited PI3K/Akt/mTOR signaling measured by decreased p-Akt and p-rpS6 expression [194,196]. However, many cells were resistant following gedatolisib treatment, which was attributed to over-activation of the MEK/ERK pathway through suppression of mTORC2 [199,200].

The clinical activity and tolerance of gedatolisib for AML therapy was evaluated in an open, prospective, single arm, multicentric Phase 2 study published by Vargaftig et al. [201]. Twelve patients were enrolled of which 10 were retained for analysis. All patients were adults diagnosed with relapsed AML (first relapse (*n* = 5), second relapse (*n* = 5)) and symptomatic uncontrolled AML. One patient had therapy-related AML (prostate adenocarcinoma). Overall, only one patient completed the treatment, and this study was terminated as no objective response was detected in any of the patients. Transcriptomic analysis revealed that gedatolisib treatment weakly affected gene expression pattern, of which four genes found to be upregulated were associated with natural killer (NK) cell immunity. This suggests that gedatolisib might affect immune cells. Current research is focusing on understanding mechanisms of resistance to gedatolisib in solid tumors.

### 4.3. Idelalisib

Idelalisib (CAL-101; GS-1101) is a highly selective inhibitor of PI3Kδ that has entered clinical trials for the treatment of hematologic malignancies. In a Phase 1 clinical trial, idelalisib was evaluated in relapsed/refractory CLL, non-Hodgkin lymphoma (NHL), AML, and multiple myeloma (MM). The results of this clinical study has been presented at several conferences and has been published in several reports [202,203,204,205]. Although it has been demonstrated that idelalisib has an acceptable safety profile and rapidly induced durable disease responses in CLL, NHL, and mantle cell lymphoma (MCL), the outcomes in AML have not yet been published. In one report, the interim results of the study were discussed in which seven AML patients were included [206]. The report suggests that idelalisib was not effective in AML.

### 4.4. Dactolisib

Dactolisib (BEZ235; NVP-BEZ235) is a dual ATP-competitive pan-class PI3K and mTOR inhibitor that has been clinically evaluated alone or in combination therapy in Phase 1/2 studies in solid tumors and hematological malignancies [207,208,209]. Several reports have demonstrated that dactolisib exerts anti-proliferative and cytotoxic effects in vitro. Dactolisib was also shown to induce autophagy and enhance chemosensitivity [210,211,212]. In AML, dactolisib showed similar biological effects in vitro and inhibited the colony-forming capability of LSCs [213,214]. Results obtained from clinical investigation of dactolisib for advanced cancers reported frequent dose-limiting toxicities accounting for adverse effects. Several Phase 2 studies reported poor tolerance of dactolisib with only modest clinical activity, which led to treatment discontinuation [208,209].

A Phase 1 study by Wunderle et al. evaluated the safety and efficacy of BEZ235 among 22 patients with relapsed or refractory AML (*n* = 11), B-cell precursor ALL (*n* = 9), T-cell ALL (*n* = 1), and chronic myeloid leukemia (CML) in myeloid blast phase (*n* = 9). Clinical responses were observed in four patients (18.2%): complete hematological and molecular remission or hematological improvement in three ALL patients and stable disease of four months in one AML patient. Notably, no activating mutations of PIK3CA, PTEN, or AKT were detected in any of the patients [108]. Detection of p-Akt, p-S6, and p-4E-BP1 by western blot and flow cytometry did not correlate with response. Similar to previous clinical findings, dactolisib induced adverse effects such as gastrointestinal implications, pneumonia, and sepsis. Due to disease progression, 19 patients (86.4%) were withdrawn from the study [215]. Together, these data raise the question whether inhibiting both PI3K and mTOR is a viable therapeutic strategy and whether we can do better.

### 4.5. MK-2206

MK-2206 is a highly specific allosteric pan-Akt inhibitor for all three isoforms of human Akt that has entered clinical trials for solid tumors as well as hematological malignancies. In vitro, MK-2206 induced apoptosis and enhanced the cytotoxicity of chemotherapy [216]. Although it was reported that MK-2206 effectively suppressed phosphorylation of Akt, MK-2206 monotherapy showed limited clinical activity, due to incomplete target inhibition at tolerable doses, paving the way for combination strategies [217,218,219].

A Phase 2 study by Konopleva et al. evaluated the clinical efficacy and tolerability of MK-2206 in 19 patients, of which 18 were retained for analysis [220]. All patients were adults with relapsed/refractory AML requiring second salvage therapy. No activating mutations of upstream RTK were detected. In preclinical studies, MK-2206 inhibited cell growth and induced apoptosis in AML cell lines and primary samples. In clinical studies, among 18 evaluable patients, only one patient (5.6%) achieved CR with incomplete platelet count recovery. The remaining 17 patients (94.4%) were withdrawn from the study due to disease progression. Proteomic analysis by RPPA following MK-2206 treatment revealed upregulation of a subset of pro-survival proteins, suggesting that other approaches to block Akt signaling should be exploited for AML.

### 4.6. Afuresertib

Afuresertib (GSK21110183) is a reversible, ATP-competitive pan-Akt inhibitor that has entered clinical trials, both as monotherapy and combination with other agents, for solid tumors and MM [221,222,223]. In vitro, afuresertib induced apoptosis and G1 phase arrest and effectively suppressed phosphorylation of multiple Akt substrates, including FoxO1, GSK3β, mTOR, and p70. Spencer et al. evaluated the maximum tolerated dose (MTD), safety, pharmacokinetics, and clinical activity of afuresertib in an open-label Phase 1 study for advanced hematological malignancies [224]. A total of 73 patents were included with MM (*n* = 34), NHL (*n* = 13), AML (*n* = 9), Hodgkin disease (*n* = 8), CLL (*n* = 7), ALL (*n* = 1), and Langerhan’s cell histiocytosis (*n* = 1). Among the 73 patients, 26 were evaluated for the dose escalation (part 1) and 47 patients in the expansion cohort (part 2). Disease inclusion criteria for part 2 were CLL, CML, ALL, AML, MM, or NHL. Clinical activity was observed primarily in MM patients and in patients with lymphomas. Overall, three patients (9%) achieved partial remission (PR), three patients (9%) achieved minimal response (MR), and some patients achieved prolonged disease stabilization. Among the nine AML patients, treatment failure was observed in five patients, and the other four did not have response assessed. Afuresertib was safe and generally well tolerated, but effects related to the gastrointestinal tract or fatigue were noted, suggesting that Akt inhibition with afuresertib may prove beneficial for the treatment of MM.

### 4.7. Uprosertib

Uprosertib (GSK2141795) is an ATP-competitive pan-Akt kinase inhibitor that has entered clinical trials alone or in combination with MEK inhibitor trametinib for the treatment of solid tumors and hematologic malignancies [225,226]. The efficacy and safety of uprosertib in combination with trametinib in patients with RAS-mutated relapsed/refractory AML was evaluated in a single-arm, open label, multi-institution Phase 2 study published by Ragon et al. [227]. In total, 23 patients were enrolled diagnosed with de novo AML (*n* = 9) or post-myelodysplastic syndromes (MDS)/myeloproliferative neoplasms (MPN)/chronic myelomonocytic leukemia (CMML) AML (*n* = 14). Molecular analysis confirmed KRAS mutations (*n* = 5), NRAS mutations (*n* = 15), or both NRAS/KRAS (*n* = 3). Concomitant mutations were identified in eight patients, including FLT3 (*n* = 1), IDH1/2 (*n* = 2), runt-related transcription factor 1 (RUNX1) (*n* = 1), DNMT3A (*n* = 3), additional sex combs like-1 (ASXL1) (*n* = 1), NPM1 (*n* = 1), CCAAT/enhancer-binding protein alpha (CEBPA) (*n* = 1), and protein tyrosine phosphatase non-receptor type 11 (PTPN11) (*n* = 2). RPPA and complementary flow cytometry analysis was performed to determine the effect of treatment on activation of PI3K/Akt/mTOR, MAPK, and other signaling pathways. RPPA analysis revealed that, in contrast to preclinical studies, no significant downregulation of PI3K/Akt/mTOR and MAPK signaling was observed following treatment with the drug combination. Phospho-flow analysis on the other hand demonstrated significant inhibition of p-ERK and a trend towards significant inhibition of p-rpS6 in peripheral blood blasts and CD34+ peripheral blood blasts. However, inhibition of MEK and Akt did not yield improved clinical efficacy. Overall, no patient obtained complete remission (CR) or CR with incomplete recovery of platelets (CRp), and the study was discontinued due to disease progression, dose-limiting toxicity (DLT), adverse effects determined unrelated to therapy, and death. Most toxicities were mild and included diarrhea, maculopapular rash, mucositis, and nausea/vomiting. Minor hematologic improvement and unconfirmed minor response was observed in seven patients. Ragon et al. suggest that this dual targeting approach could prove to be an effective treatment strategy in combination with cytotoxic, targeted, or epigenetic therapies to overcome possible compensatory signaling.

### 4.8. Sirolimus

Sirolimus, also known as rapamycin, is a naturally occurring macrolide compound that interacts with FKBP12. This complex binds to the FKBP rapamycin binding (FRB) domain located in the carboxyl terminus of mTOR and inhibits its kinase activity. In vitro, sirolimus inhibits cancer cell growth, promotes G1 cell cycle arrest, and enhances cytotoxic effects in combination with other agents [228,229,230,231]. Sirolimus is clinically often used in renal transplantations and for the treatment of a rare lung disease called lymphangioleiomyomatosis (LAM) but has also been investigated for the treatment of solid tumors and hematological malignancies [184,232,233,234,235].

Kasner et al. evaluated the clinical potential of sirolimus for AML therapy in two sequential clinical trials in identical patient populations (NCT00780104 and NCT01184898). In total, 52 patients were enrolled, with 51 undergoing clinical evaluation. Among the 51 patients clinically evaluated, the majority had abnormal cytogenetics (56%), and FLT3-ITD was detected in seven patients (14%). The pilot study provided evidence that intracellular flow cytometry can be applied to monitor the biochemical efficacy of drugs targeting the PI3K/Akt/mTOR signaling pathway. Results revealed heterogeneity of rpS6 phosphorylation among particular patient populations, suggesting that evaluation of drug sensitivity ex vivo or in early therapy may prove useful in predicting response to molecular targeted therapies [236,237]. In the final clinical and pharmacodynamics study, the clinical response of sirolimus in combination with mitoxantrone/etoposide/cytarabine (MEC) chemotherapy in a larger study cohort was evaluated. Overall, 24 patients responded (47%) with 18 CR (35%), one CRp, and five PRs. Sirolimus combination with MEC was tolerable in patients with high risk AML; however, the combination therapy did not enhance clinical responses in AML and was limited to patients presenting baseline mTORC1 activation as measured by baseline rpS6 phosphorylation. [238,239].

### 4.9. Everolimus

Everolimus (RAD001), a derivative of rapamycin, is an allosteric inhibitor of mTORC1 showing promise for several cancer types. Everolimus was granted FDA approval for progressive, well-differentiated non-functional, neuroendocrine tumors of gastrointestinal or lung origin with unresectable, locally advanced, or metastatic disease [182]. Everolimus interacts with FKBP12, affecting downstream effectors of mTORC1 and ultimately inhibiting cell proliferation [240,241]. However, rapamycin-induced targeting of mTORC1 has not been effective inhibiting PI3K/Akt/mTOR activity, which may relate to a negative feedback loop promoting paradoxical hyperactivation of Akt, insensitivity to rapamycin, and IGF-1-mediated feedback loops [242,243].

A Phase 1b study by Park et al. evaluated the efficacy and tolerance of everolimus combined with conventional chemotherapy (daunorubicin plus cytarabine) in 28 younger (<65 years old) AML patients at first relapse [244]. Karyotyping revealed that six patients (21.4%) had poor cytogenetic abnormalities, 19 patients (67.9%) intermediate risk, and three (10.7%) good prognostic cytogenetic indicators. Mutational analysis identified FLT3-ITD or NPM1 mutation in each of two patients and co-existence of FLT3-ITD and NPM mutation in three patients (10.7%). Everolimus strongly inhibited mTORC1 signaling measured by decreased p-p70S6K at T389. Clinical outcomes revealed that 19 patients (67.9%) achieved CR. Notably, three out of four patients with FLT3-ITD achieved CR. Overall, this study showed that a weekly dose of everolimus (70mg) in combination with conventional therapy was tolerable and had acceptable toxicity in younger AML patients at relapse.

Another Phase 1b study by Tiong et al. evaluated the efficacy and tolerability of everolimus in combination with low dose cytarabine (LDAC) in elderly AML [245]. Twenty-four AML patients diagnosed with de novo AML (*n* = 15) or secondary AML (*n* = 9) were included. Mutational analysis by multiplexed mass spectrometry detected mutation of NRAS/KRAS (*n* = 4), NPM1 (*n* = 3), IDH1/2 (*n* = 3), DNMT3A (*n* = 2), FLT3 D835 (*n* = 2), MPL (*n* = 1), and FLT3-ITD (*n* = 1). Overall response rate (ORR) was achieved in six patients (25%) with four CR or CR with incomplete hematological recovery (CRi) (16.7%), one PR (4.2%), and one morphologic leukemia-free state (4.2%). Furthermore, two patients (8.3%) had >50% reduction in marrow leukemic blasts without blood count recovery. However, there was no clear association between mutational profile and response despite two responding patients carrying a *RAS* mutation. The combination of everolimus with LDAC neither revealed an efficacy signal nor potentially increased toxicity. The authors suggested combining mTOR-directed therapies with other novel agents for the treatment of AML.

## 5. Clinical Strategies to Overcome Resistance to PI3K/Akt/mTOR Inhibitors in AML

**Figure 4:**
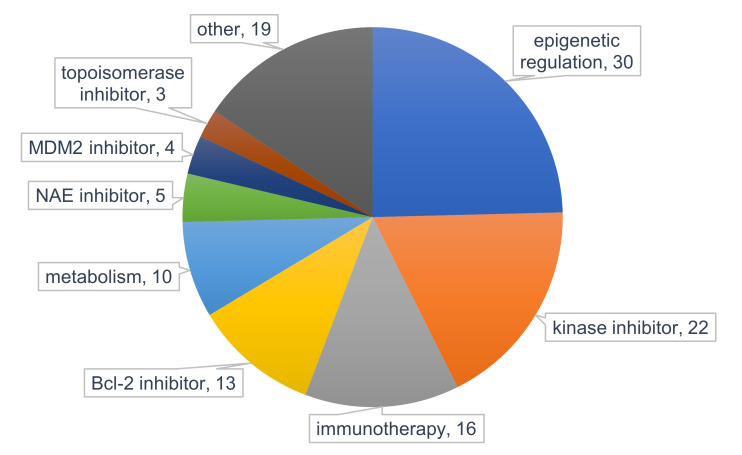
Pie chart presenting the classification of interventions for AML therapy evaluated in clinical trials. Data was obtained from ClinicalTrials.gov and filtered for “recruiting” and “active, not recruiting” status in adult and elderly AML and study phase (1b-3) with start date from 1 January 2018. Numbers present frequency within 88 studies. * other = frequency <3 include dihydroorotate dehydrogenase (DHODH) inhibitor, proteasome inhibitor, p53 activator, hedgehog signaling inhibitor, CDK inhibitor, E-selectin inhibitor, nuclein export inhibitor, menin-MLL binding interaction inhibitor, CXCR4 antagonist, proteasome inhibitor, corticosteroid, thrombopoietin receptor agonist, and isoprenyl transferase inhibitor. MDM2 = mouse double minute 2 homolog, NAE = NEDD8-activating enzyme, Bcl-2 = B-cell lymphoma 2.

In the past, PI3K/Akt/mTOR inhibitors have been tried mostly combined with cytotoxic chemotherapeutics to improve efficacy and reduce drug-related toxicity. However, combination with targeted therapies may be more effective and cooperatively inhibit PI3K/Akt/mTOR signaling while preventing escape via alternative pathways [246]. Several new, targeted agents are currently under clinical investigation for AML therapy, including drugs targeting epigenetic regulators, kinases, monoclonal antibodies, Bcl-2, and metabolism (Figure 4). Here, we will provide the rationale for clinically investigating potential drug combinations using PI3K/Akt/mTOR inhibitors as a novel targeted therapeutic strategy for AML.

### 5.1. PI3K/Akt/mTOR Inhibitors in Combination with Epigenetic Targeting

The importance of epigenetics in AML pathology was demonstrated by the identification of recurrent mutations in genes encoding epigenetic regulators, such as DNA methyltransferase 3A (DNMT3A), Tet methylcytosine dioxygenase 2 (TET2), and IDH1/2 [247]. In addition to mutations in epigenetic regulators, oncogenic fusion proteins can dysregulate epigenetic modifications, driving abnormal transcriptional programs in AML [248,249]. Several targeted epigenetic therapies have been explored for AML, for example, hypomethylating agents (HMA), histone deacetylase (HDAC) inhibitors, and IDH1/2 inhibitors. However, as monotherapy these inhibitors do not seem to be effective, and treatment failure is common [250,251,252,253]. To potentiate the efficacy of epigenetic therapy, combination strategies with cytotoxic chemotherapy or other targeted drugs are currently being extensively studied.

Epigenetic modifiers participate in the PI3K/Akt/mTOR pathway, contributing to the oncogenicity of PI3K in cancer. The cooperation of the PI3K/Akt/mTOR pathway with epigenetic reprogramming involves modulation of DNA methylation and histone methylation to promote transcriptional competence [254,255]. Because of this collaboration, combination strategies with PI3K/Akt/mTOR inhibitors and epigenetic therapies have been exploited. There is emerging evidence demonstrating synergism when these classes of agents are combined, involving Akt inactivation, FoxO3a-mediated upregulation of pro-apoptotic targets Bim and Puma, and Mcl-1 downregulation [256,257,258,259]. PI3K/Akt/mTOR inhibitors may potentiate epigenetic therapies inducing LSC death by enhanced activation of ROS, to damage DNA [259,260]. In addition to enhanced anti-leukemic effects, there is preclinical evidence suggesting that DNMT or HDAC inhibitors can overcome acquired resistance to PI3K/Akt/mTOR inhibitors [261,262].

To our knowledge, there are two clinical studies that have evaluated the combination of mTOR inhibitors with a hypomethylating agent, reported by Liesveld et al. and Tan et al. Liesveld et al. evaluated decitabine followed by sirolimus in an open label, single arm, Phase 1 dose escalation study in relapsed/refractory AML [263]. Decitabine is a DNA-hypomethylating agent that has been reported to markedly improve CR rates compared to cytarabine but failed to significantly improve OS rates [264,265]. Thirteen patients were subjected to therapy of which 12 were included for safety evaluation. Complex cytogenetics were present in most patients and only one patient carried the FLT3-ITD mutation at diagnosis. Overall, the administration of decitabine followed by sirolimus was well tolerated and among the evaluable patients, but only one patient achieved CR. Four patients (31%) demonstrated disease progression, 5 (38%) had stable blast percentage, and 4 (31%) demonstrated reduced blast percentage after one cycle. Usually multiple cycles are required to achieve clinical response, but this was not possible in many of the patients due to persistent cytopenias and need for more palliative approaches. Furthermore, the response to inhibition of PI3K/Akt/mTOR signaling was inconsistent, reflecting on the heterogeneous nature of AML. More potent PI3K/Akt/mTOR inhibitors may be more effective in these studies.

Tan et al. evaluated the maximum tolerated dose (MTD) and efficacy of everolimus, a more potent mTOR inhibitor than sirolimus, in combination with azacitidine subcutaneously in an open-label, Phase 1b/2 study in relapsed/refractory AML. Forty patients with relapsed (*n* = 27), primary refractory (*n* = 11) or elderly patients unfit for intensive chemotherapy (*n* = 2) were enrolled, of which 37 were retained for evaluation of toxicity [266]. Similar to Liesveld et al., azacitidine and everolimus were given sequentially to maximize mTOR inhibitory effect and avoid potential mTOR-induced cell cycle inhibition which may attenuate azacitidine activity. Mutational analysis revealed IDH1/2 mutations in 15/35 (43%) and FLT3-ITD mutation in 7/37 (19%) patients. Notably, three patients with relapsed FLT3-ITD had >80% bone marrow blast reductions following sequential therapy, of which two underwent allogeneic stem cell transplantation but subsequently relapsed and died. Overall, the combination of everolimus and azacitidine was well tolerated with an ORR of 22.5% and mean survival of 8.5 months. Collectively, these findings support the clinical investigation of PI3K/Akt/mTOR inhibitors in combination with targeted epigenetic therapies in AML patients.

### 5.2. PI3K/Akt/mTOR Inhibitors in Combination with Bcl-2 Inhibitors

Bcl-2, encoded by the human *BCL2* gene is the founding member of the Bcl-2 family of proteins which are divided into pro-apoptotic and anti-apoptotic members. The anti-apoptotic proteins include Bcl-2, Bcl-XL, Bcl-w, Mcl-1, and A1 that share three or four sequence homologies in the Bcl-2 homology (BH) domain. These proteins can directly interact with pro-apoptotic BH3-only proteins Bim, Puma, Bad, Bid, Bik, Bmf, Hrk, and Noxa, which share homology in the BH3 domain [267]. Apoptotic stimuli upregulate BH3-only proteins Bax and Bak that lead to permeabilization of the mitochondrial outer membrane and release cytochrome c into the cytosol for activation of caspases that dismantle cellular structures [268,269,270].

Bcl-2 is often overexpressed in AML patients, both at diagnosis as well as at relapse and can render resistance to cytotoxic chemotherapeutics [271,272]. A major finding by Lagadinou et al. identified high levels of Bcl-2 as a feature of LSCs to maintain mitochondrial integrity for survival [27]. LSCs were characterized by low levels of ROS and displayed overexpression of Bcl-2. Importantly, it was demonstrated that small molecule Bcl-2 inhibitors reduced oxidative phosphorylation and selectively killed quiescent LSCs. Thus, targeting Bcl-2 may provide a promising therapeutic strategy to effectively target chemoresistant LSC population. BH3 mimetic venetoclax (ABT-199) is a specific inhibitor of Bcl-2 and has been approved by the FDA for the treatment of CLL. Venetoclax binds to Bcl-2 and prevents pro-apoptotic proteins Bak and Bax from binding to Bcl-2. As venetoclax showed potential both in vitro and xenograft models, its efficacy was evaluated for AML [273,274]. Konopleva et al. evaluated the efficacy of venetoclax as monotherapy in a Phase 2 study in 32 patients with high-risk relapsed/refractory AML or unfit for intensive chemotherapy [275]. Although venetoclax had acceptable tolerability, the ORR was 19%, of whom 6% achieved CR and 13% achieved CRi. Unfortunately, the clinical response of venetoclax was short-lived as all patients relapsed, suggesting the rapid development of venetoclax resistance. Myoblast dependence on Mcl-1 and Bcl-xL were identified as key players in the intrinsic resistance to venetoclax in AML [275,276]. In contrast to CLL, which is relatively homogenously dependent on Bcl-2, AML seems more heterogeneous and depends on multiple pro-apoptotic proteins [277,278]. Significant efforts have been made to overcome this resistance by combining venetoclax with DNA damaging agents such as daunorubicin and cytarabine, which reduce Mcl-1 levels and enhance venetoclax activity [279,280]. Hypomethylating agent azacitidine was also shown to reduce Mcl-1 protein levels, and synergistically induced apoptosis in AML cells [281]. The FDA approved venetoclax in combination with HMAs or LDAC for the treatment of AML patients who are previously untreated and older or unfit for chemotherapy [282,283,284].

In addition to cytotoxic chemotherapy and hypomethylating agents, PI3K/Akt/mTOR inhibitors may also overcome venetoclax resistance and potentiate LSC targeting. The combination of PI3K/Akt/mTOR inhibitors with Bcl-2 inhibitors has been examined in several preclinical cancer models [285,286]. Results demonstrated that PI3K/Akt/mTOR inhibitors downregulate Mcl-1, in part through GSK3 activation, and upregulate Bim, Bad, Bax and Bak. Although PI3K/Akt/mTOR inhibition downregulated Mcl-1, it did not trigger apoptosis, presumably by increased Bim binding to Bcl-2 and Bcl-XL. Co-targeting Bcl-2 released Bim from Bcl-2 and Bcl-xL and induced Bax and Bak-dependent apoptosis in AML LSCs, whilst sparing normal hematopoietic progenitor cells [28,287,288,289,290]. These data support the rationale for the evaluation of the combination of PI3K/Akt/mTOR inhibitors and Bcl-2 inhibitors in AML. Currently, there is one study exploring the combination of pan-PI3K inhibitor copalisib and venetoclax in relapsed/refractory NHL (NCT03886649) [291].

### 5.3. PI3K/Akt/mTOR Inhibitors in Combination with Kinase Inhibitors

Previously, we discussed other signaling pathways that can integrate with the PI3K/Akt/mTOR pathway, which can potentially reduce the activity of PI3K/Akt/mTOR inhibitors. Parallel pathway inhibition may improve sensitivity to PI3K/Akt/mTOR inhibitors and enhance anti-leukemic effects in AML cells. For example, upregulation of PIM was shown to cause resistance to PI3K/Akt/mTOR inhibitors in AML through a mechanism that involves modulation of mTORC1 activity [158,292]. While pan-PIM inhibitor AZD1897 showed limited activity as monotherapy in AML cell lines and primary samples, including FLT3-ITD+ cells, combination of AZD1897 with Akt inhibitor AZD5363 displayed synergistic anti-leukemic activity [293]. This was associated with enhanced inhibition of mTORC1 signaling activity and Mcl-1 levels. Importantly, this combination strategy affected both bulk cells and LSC subsets.

There are two studies in which PIM inhibitors and PI3K inhibitors have been evaluated as combination therapy for hematological malignancies. Novartis Pharmaceuticals evaluated the safety and effectiveness of pan-PIM inhibitor LGH447 and PI3Kα inhibitor BYL719 in patients with relapsed/refractory MM in a Phase 1b/2 study, but the trial never made it to Phase 2 (NCT02144038). Incyte Corporation is currently examining the combination of NCB053914 (pan-PIM inhibitor) with INCB050465 (PI3Kδ inhibitor) in relapsed/refractory diffuse large B-cell lymphoma (DLBCL) in a Phase 1b study (NCT03688152) [294].

Co-Targeting the PI3K/Akt/mTOR and MAPK/ERK pathways is another combination strategy that has been evaluated. In AML, the FLT3-ITD mutation is an important mechanism that feeds growth signals into main oncogenic signaling pathways such as PI3K/Akt/mTOR and MAPK/ERK. As crosstalk exists between PI3K/Akt/mTOR and MAPK/ERK pathways, inactivation of both pathways may interrupt oncogenic signals in AML [126,295]. This may be particularly beneficial in RAS mutated cases. In fact, several reports have shown in vitro enhanced induction of apoptosis when PI3K/Akt/mTOR inhibitors were combined with MAPK/ERK inhibitors [296]. Inhibition of MEK suppressed p-Erk level but caused feedback activation of the PI3K/Akt/mTOR pathway defined by increased p-mTOR and Mcl-1 levels which could be abrogated by co-targeting PI3K/Akt/mTOR [297]. The MEK inhibitor also resulted in increased expression of Bim and Bcl-2, which could not be abrogated by adding the PI3K/Akt/mTOR inhibitor. It has been suggested that the increased Bim was bound to Bcl-2, which prevented induction of cell death, and that inhibition of Bcl-2 might further sensitize AML cells to apoptotic cell death by the combination of the MEK and PI3K/Akt/mTOR inhibitors [287]. Indeed, simultaneous inhibition of PI3K, mTOR, and MAPK/ERK synergistically induced cell death in AML cells, which was further enhanced by the addition of venetoclax, while sparing the normal hematopoietic progenitor cells [287,297]. This triple-drug combination may potentiate the efficacy of venetoclax to target quiescent LSCs. There is one Phase 1b study that examined the MTD for the combination of PI3Kα inhibitor BYL719 and allosteric MEK1/2 inhibitor MEK162 in patients with advanced solid tumors and in adult patients with AML or high risk MDS carrying RAS or BRAF mutations (NCT01449058). However, the preliminary safety and efficacy findings warrant further exploration as adverse effects were common and stable disease lasting >6 weeks was noted as best response for 31% of patients [298].

Furthermore, dual inhibition of FLT3 activation and downstream targeting of PI3K/Akt/mTOR may have synergistic activity in FLT3 mutated cases. Patients with FLT3-ITD tend to have a high risk of relapse and shorter OS compared to patients without the mutation [14]. In the last few years, several FLT3 tyrosine kinase inhibitors have been developed, which can be divided into first-generation pan-kinase inhibitors such as sorafenib and midostaurin, and next generation inhibitors such as quizartinib and gilteritinib, which are more potent and specific for FLT3 [299]. Although FLT3 inhibitors were able to induce response in AML patients with FLT3 mutations, these responses were often not durable, and resistance developed rapidly [300,301]. FLT3 inhibitors are currently evaluated in combination with chemotherapy or hypomethylating agents in various settings, to overcome resistance and prolong OS, respectively [300,302,303]. The PI3K/Akt/mTOR pathway is a promising target to overcome resistance to FLT3 inhibitors. Upregulation of PI3K/Akt/mTOR pathway was shown in FLT3 inhibitor-resistant FLT3-ITD+ AML cells despite inhibition of FLT3 activation by FLT3 inhibitors, suggesting that the resistant cells become FLT3 independent [304,305]. Inhibition of PI3K/Akt/mTOR in vitro improved sensitivity to FLT3 inhibition and enhanced inhibition of growth and apoptosis. PI3K/Akt/mTOR inhibitors may also potentially suppress FLT3-ITD-induced LSC survival. It has been reported that FLT3-ITD upregulates Mcl-1 to promote survival of AML LSCs, which was abrogated by the tyrosine kinase inhibitor midostaurin (PKC-412) that targets multiple receptors (including FLT3 and c-Kit) [157]. As PI3K/Akt/mTOR inhibitors can induce inhibition of Mcl-1, combination of PI3K/Akt/mTOR inhibitors with FLT3 inhibitors may potentiate inhibition of LSC survival. Currently, there is one Phase 1 study evaluating the combination of mTOR inhibitor everolimus with midostaurin in patients with relapsed/refractory or poor prognosis AML or MDS (NCT00819546).

### 5.4. PI3K/Akt/mTOR Inhibitors in Combination with DNA Repair Inhibitors

In a previous section, we briefly highlighted the importance of increased ROS in AML, which can promote genomic instability and contributes to chemotherapy resistance. In particular, there is increasing evidence associating FLT3 mutations with DNA damage, specifically through increased ROS, resulting in double strand breaks (DSBs) and impaired DNA repair [132,306]. Targeting DNA damage repair mechanisms may provide a novel therapeutic opportunity in AML that may reduce repair errors and genomic instability [307]. Genomic stability is maintained by the DDR, a highly orchestrated signaling cascade that results from the induction and detection of DNA damage. At the heart of the DDR are members of the PIKK family comprising ataxia telangiectasia mutated (ATM), ataxia telangiectasia and Rad3-related protein (ATR), and DNA-PK, which upon sensing of DNA damage, in particular DSBs, phosphorylate a large number of substrates to effectively repair DNA [308]. Although DSB repair in normal mammalian cells is primarily dominated by homologous recombination (HR) and DNA-PK-dependent NHEJ, FLT3-ITD directs DSB repair towards an error-prone, alt-NHEJ pathway, which is mediated by poly(ADP-ribose) polymerase-1 (PARP-1) and DNA Lig IIIα [309,310].

In this regard, PARP inhibitors present a putative therapeutic strategy for AML. PARP1, an important protein of the DDR pathway, senses single strand breaks (SSBs) and has a crucial role for base excision repair (BER). Inhibition of PARP1 leads to accumulation of SSBs which then become DSBs that make cells more dependent on HR [311,312]. However, in cells with defective HR, such as those with BRCA deficiency, PARP1 inhibition induced synthetic lethality [313,314]. This concept of synthetic lethality using PARP inhibitors has been effectively examined in BRCA1-defective solid tumors, but was also exploited for the treatment hematological malignancies like AML [315,316,317]. Indeed, several in vitro studies in AML reported that PARP inhibitors induced cell cycle arrest and apoptosis, and combination with chemotherapy may induce synthetic lethality [133,318,319].

There is ongoing research evaluating the combination of PARP inhibitors with other agents to extend the therapeutic potential of these inhibitors beyond tumors with mutations of BRCA or other HR deficiencies. As the PI3K/Akt/mTOR pathway contributes to the DDR, combination of PI3K/Akt/mTOR inhibitors with PARP inhibitors was exploited in several solid tumor models [320,321]. Inhibition of the PI3K/Akt/mTOR pathway in ovarian, breast, and prostate cancers revealed impaired expression of BRCA1/2 and reduced cellular capacity to conduct HR [322,323,324,325]. Consistent with the reduction in the expression of BRCA1/2, PI3K/Akt/mTOR inhibitors sensitized cells to PARP inhibition and synergistically suppressed cell growth. Although these findings support the rationale for the combination of PI3K/Akt/mTOR inhibitors with PARP inhibitors, this combination strategy is yet to be evaluated in AML.

## 6. Conclusions and Future Directions

The PI3K/Akt/mTOR pathway is central to a broad range of cellular regulatory processes, such as proliferation, differentiation, and survival. Dysregulation of this pathway is common in AML and is often caused by mutations in membrane-bound proteins—in particular FLT3-ITD. Constitutive activation of PI3K/Akt/mTOR induced by such activating mutations is associated with poor OS and chemoresistance. As the PI3K/Akt/mTOR pathway is considered a putative target for AML, significant efforts have been made to develop small-molecule drugs that showed promise in preclinical settings. However, the initial clinical results using PI3K/Akt/mTOR inhibitors as monotherapy have not been successful in clinical practice due to drug-related toxicity, incomplete pathway inhibition, and heterogeneous constitutive activation of the pathway. Therefore, PI3K/Akt/mTOR inhibitors may be more effective in combination with other therapeutic agents. Several clinical studies have exploited the combination of PI3K/Akt/mTOR inhibitors with induction chemotherapy, and although this drug combination strategy was generally well tolerated with acceptable toxicities, only modest improvement of clinical outcome was achieved. Notably, activation of parallel pathways that cooperate with PI3K/Akt/mTOR was reported, and therapeutic targeting of the PI3K/Akt/mTOR pathway was restricted to only a subset of patients. Given the limited clinical effects of PI3K/Akt/mTOR inhibitors as monotherapy or in combination with induction chemotherapy, it appears more promising to evaluate the combination of PI3K/Akt/mTOR inhibitors with other agents, e.g., novel targeted therapies or pathway inhibitors. In that regard, we have discussed how other signaling pathways impinge on the PI3K/Akt/mTOR signaling pathway activity and provided rationale for combining PI3K/Akt/mTOR inhibitors with novel targeted agents that have emerged as potential therapeutic candidates for AML. These combination treatment strategies may effectively inhibit the PI3K/Akt/mTOR pathway and kill AML cells. It is however important to identify the appropriate subgroups of patients that would benefit from such drug combination strategies.

## Figures and Tables

**Table 1:** PI3K and mTOR inhibitors approved by FDA for human cancers.

Compound	Manufacturer	Generic Name	Year	Target	Disease/Condition	Reference
Copiktra^®^	Verastem Oncology, Needham, MA, USA	duvelisib	2018	PI3K-δ PI3K-γ	Relapsed or refractory CLL, SLL, FL	[30]
Aliqopa^®^	Bayer HealthCare Pharmaceuticals Inc., Whippany, NJ, USA	copanlisib	2017	PI3K-α PI3K-δ	Relapsed FL	[29]
Zydelig^®^	Gilead Sciences, Foster City, CA, USA	idelalisib	2014	PI3K-δ	Relapsed CLL in combination with rituximab, relapsed FL and SLL	[31]
Piqray^®^	Novartis, Basel, Switzerland	alpelisib	2019	PI3K-α	In combination with fulvestrant; hormone receptor (HR)-positive, human epidermal growth factor receptor 2 (HER2)-negative, PIK3CA-mutated, advanced or metastatic breast cancer	[181]
Afinitor^®^	Novartis, Basel, Switzerland	everolimus	2009	mTOR	Progressive, well-differentiated non-functional, NET of GI or lung origin with unresectable, locally advanced or metastatic disease	[182]
Torisel^®^	Pfizer Inc., New York City, NY, USA	temsirolimus	2007	mTOR	Advanced renal cell carcinoma	[183]
Rapamune^®^	Pfizer Inc., New York City, NY, USA	sirolimus	1999	mTOR	LAM, kidney transplant	[184]

CLL = chronic lymphocytic leukemia, SLL = small lymphocytic lymphoma, FL = follicular lymphoma, HER = human epidermal growth factor receptor, NET = neuroendocrine tumors, GI = gastrointestinal, LAM = lymphangioleiomyomatosis.

**Table 2:** Phase 1/2 clinical trials using PI3K/Akt/mTOR inhibitors.

Investigational Agent	Condition	Phase	Status	Subjects	Study Period	Trial Number
gedatolisib	Therapy-related AML and MDS	2	Terminated	10	2015–2018	NCT02438761
buparlisib	Advanced Leukemias	1	Completed	16	2012–2016	NCT01396499
idelalisib	CLL, NHL, AML, MM	1	Completed	192	2008–2012	NCT00710528
Personalized kinase inhibitor therapy (dasatinib, idelalisib, ruxolitinib, ponatinib, sorafenib, sunitinib) + chemotherapy +/− monoclonal antibody treatment: (cyclophosphamide, cytarabine, doxorubicin, idarubicin, methotrexate, vincristine, rituximab)	ALL, AML	1	Recruiting	24	2016–2019	NCT02779283
dactolisib	ALL, AML, CLL with crisis of blast cells	1	Unknown	23	2017–2017	NCT01756118
MK-2206	Relapsed or refractory AML	2	Completed	19	2010–2018	NCT01253447
afuresertib	Hematological malignancies	1/2	Completed	73	2009–2012	NCT00881946
uprosertib + trametinib	Recurrent or untreated adult AML	2	Terminated	24	2013–2018	NCT01907815
everolimus + midostaurin	AML, MDS	1	Active, not recruiting	29	2009–	NCT00819546
everolimus + nilotinib	AML	1/2	Completed	40	2008–2012	NCT00762632
everolimus + cytarabine/daunorubicin	AML	1	Completed	31	2010–2012	NCT01074086
everolimus + cytarabine	AML	1	Unknown	40	2008–	NCT00636922
everolimus + cytarabine/daunorubicin	Relapsed AML	1	Unknown	21	2007–	NCT00544999
sirolimus + MEC	AML, myeloid leukemias, leukemia, CML	1	Completed	16	2007–2010	NCT00780104
sirolimus + MEC	AML	NA	Completed	36	2010–2016	NCT01184898
sirolimus + idaurubicin/cytarabine	AML, PEL	1	Completed	55	2013–2019	NCT01822015
sirolimus + azacitidine	(Recurrent) AML, de novo MDS, MDS with isolated del(5q) previously treated MDS	2	Active, not recruiting	57	2013–	NCT01869114
sirolimus + decitabine	Relapsed or refractory AML	1	Completed	13	2009–2012	NCT00861874

AML = acute myeloid leukemia, MDS = myelodysplastic syndromes, CLL = chronic lymphocytic leukemia, NHL = non-Hodgkin lymphoma, MM = multiple myeloma, ALL = acute lymphocytic leukemia, PEL = pure erythroid leukemia, CML = chronic myeloid leukemia, MEC = mitoxantrone/etoposide/cytarabine; +/− = with or without.

**Table 3:** Response criteria in AML.

Response	Criteria
Complete remission (CR)	Bone marrow blasts <5%; absence of circulating blasts and blasts with Auer rods; absence of extramedullary disease; ANC ≥1.0 × 10^9^/L (1000/µL); platelet count ≥100 × 10^9^/L (100.000/µL)
CR with incomplete hematological recovery (CRi)	All CR criteria except for residual neutropenia (<1.0 × 10^9^/L [1000/µL]) or thrombocytopenia (<100 × 10^9^/L [100.000/µL])
Partial remission (PR)	All hematologic criteria of CR; decrease of bone marrow blast percentage to 5 to 25%; and decrease of pre-treatment bone marrow blast percentage by ≥50%

ANC = absolute neutrophil count.
